# Gut microbiota functions: metabolism of nutrients and other food components

**DOI:** 10.1007/s00394-017-1445-8

**Published:** 2017-04-09

**Authors:** Ian Rowland, Glenn Gibson, Almut Heinken, Karen Scott, Jonathan Swann, Ines Thiele, Kieran Tuohy

**Affiliations:** 1 Department of Food and Nutritional Sciences, University of Reading, Whiteknights, Reading, RG6 6AP UK; 2 Luxembourg Centre for Systems Biomedicine, University of Luxembourg, Campus Belval, Avenue des Hauts Fourneaux 7, 4362 Esch-sur-Alzette, Luxembourg; 3 The Rowett Institute, University of Aberdeen, Foresterhill, Aberdeen, AB25 2ZD UK; 4 Division of Computational and Systems Medicine, Department of Surgery and Cancer, Imperial College London, London, SW7 2AZ UK; 5 Nutrition and Nutrigenomics Unit, Department of Food Quality and Nutrition, Research and Innovation Centre, Fondazione Edmund Mach, Via E. Mach, 1, Trento, 38010 Italy

**Keywords:** Gut microbiota, Microbiome, Microbial metabolism, Food components, Methodology

## Abstract

The diverse microbial community that inhabits the human gut has an extensive metabolic repertoire that is distinct from, but complements the activity of mammalian enzymes in the liver and gut mucosa and includes functions essential for host digestion. As such, the gut microbiota is a key factor in shaping the biochemical profile of the diet and, therefore, its impact on host health and disease. The important role that the gut microbiota appears to play in human metabolism and health has stimulated research into the identification of specific microorganisms involved in different processes, and the elucidation of metabolic pathways, particularly those associated with metabolism of dietary components and some host-generated substances. In the first part of the review, we discuss the main gut microorganisms, particularly bacteria, and microbial pathways associated with the metabolism of dietary carbohydrates (to short chain fatty acids and gases), proteins, plant polyphenols, bile acids, and vitamins. The second part of the review focuses on the methodologies, existing and novel, that can be employed to explore gut microbial pathways of metabolism. These include mathematical models, omics techniques, isolated microbes, and enzyme assays.

## Introduction

The human colonic microbiota is a large and complex microbial community. In total, over 1000 bacterial species have been identified of which many remain uncultured, with about 160 species being found in the gut of any individual [1]. The gene set of the gut microbiota (the gut microbiome) is estimated to be about 3 million genes −150 times larger than that of the human genome [2]. This large and diverse microbial community has an equally extensive metabolic repertoire that complements the activity of mammalian enzymes in the liver and gut mucosa [3]. The gut microbiota makes an important contribution to human metabolism by contributing enzymes that are not encoded by the human genome, for example, the breakdown of polysaccharides, polyphenols and synthesis of vitamins. The evidence for the role of the microbiota in metabolism of dietary components and for its impact on health is derived from comparative studies in germ-free and conventional microbiota, or human microbiota-associated animals, and from in vitro studies using human faecal incubations or more complex continuous culture gut models. Furthermore, observational studies comparing the faecal microbiota of healthy subjects with those of patients strongly suggest that the gut microbiota plays a significant role in the aetiology and/or development of a range of gastrointestinal diseases and conditions such as inflammatory bowel disease (IBD), irritable bowel syndrome (IBS), colon cancer, and antibiotic-associated diarrhoea. More recently evidence has been accumulating that the microbiota may also be involved in obesity and diabetes [4, 5].

The critical role that the gut microbiota appears to play in human metabolism and health has stimulated research to identify the microorganisms involved and their functionality, in relation to metabolic pathways, particularly those associated with metabolism of dietary components. These areas are the focus of the present review.

## Carbohydrates

**Fig. 1:**
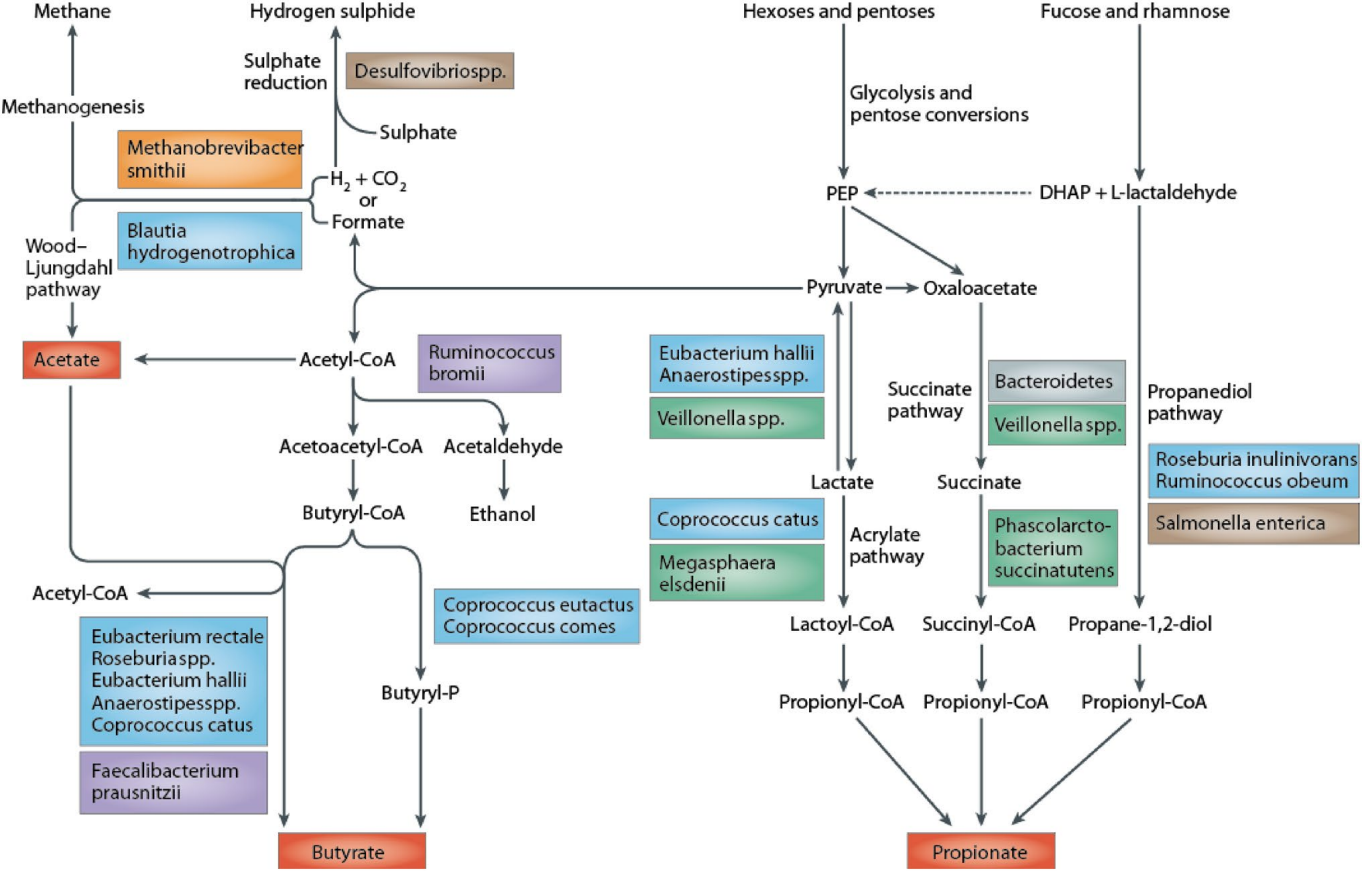
Pathways of carbohydrate metabolism [156]

Bacteria in the large intestine mainly rely on dietary substrates that are undigested in the upper digestive tract for survival. Saccharolytic bacterial fermentation produces generally beneficial metabolites, whereas if there is limited carbohydrate, bacteria turn to alternative energy sources resulting in the production of other metabolites that may be more detrimental to human health [6]. The key bacterial fermentation products following the fermentation of dietary carbohydrates are short chain fatty acids and gases (Fig. 1).

### Short chain fatty acids (SCFAs)

The three most abundant SCFAs detected in faeces are acetate, propionate, and butyrate, normally present in molar ratios ranging from 3:1:1 to 10:2:1. This ratio is consistent with values observed within the intestine in early sudden death victims [7]. These three main SCFAs perform very different but important roles in the human body. Butyrate is arguably the most important SCFA for human health. It forms the key energy source for human colonocytes and also has potential anti-cancer activity via the ability to induce apoptosis of colon cancer cells and its ability to regulate gene expression by inhibiting histone deacetylases [8]. There is also evidence that butyrate can activate intestinal gluconeogenesis (IGN) via a cAMP-dependent mechanism with beneficial effects on glucose and energy homeostasis [9]. Propionate is also an energy source for the epithelial cells, but is also transferred to the liver where it also plays a role in gluconeogenesis. It is also increasingly thought to be an important molecule in satiety signalling due to interaction with the gut receptors (G protein-coupled receptor, GPR) GPR 41 and GPR 43, also known as Fatty Acid Receptors FFAR2 and FFAR3, which may, in turn, activate intestinal IGN [9–11]. The conversion of propionate to glucose in intestinal gluconeogenesis directly promotes energy homeostasis by reducing the production of hepatic glucose, and consequently reduces adiposity [9]. Acetate is the most abundant SCFA, and is an essential co-factor/metabolite for the growth of other bacteria. For instance, *Faecalibacterium prausnitzii* will not grow in pure culture in the absence of acetate [12]. Within the human body, acetate is transported to the peripheral tissues and used in cholesterol metabolism and lipogenesis, and recent evidence from studies in mice indicates that it also plays a significant role in central appetite regulation [13].

#### Bacterial cross-feeding

Bacteria produce intermediate fermentation products including fumarate, succinate, and lactate, but these are normally detected at low levels in faeces from healthy individuals due to extensive utilization of them by other bacteria. For example, lactate is typically converted into either propionate or butyrate by other bacteria, and is thus present at negligible levels in adult faeces. However, in patients with ulcerative colitis, lactate can be detected in significantly higher amounts [14] and could potentially be an indicator of disease. Co-culture cross-feeding studies illustrate the impact of bacterial interactions on final SCFA detection. Lactate produced by *Bifidobacterium longum* during growth in pure culture on fructo-oligo-saccharides (FOS) completely disappeared in co-culture with *Eubacterium hallii*, and was replaced by significant butyrate levels- despite the fact that *E. hallii* alone could not grow on the carbohydrate substrate [15]. Growth of *Roseburia intestinalis* is stimulated by acetate and in co-culture with a different strain of *B. longum*, growth of the *R. intestinalis* on FOS was delayed until sufficient acetate, produced by *B. longum*, accumulated in the growth medium [16].

#### Specificity of SCFA production by intestinal species

Acetate is produced by many bacteria, but propionate and butyrate tend to be produced by specific bacteria [17, 18;]. Within the gastrointestinal environment, the predominant butyrate producers are Firmicutes including some Lachnospiraceae and also *Faecalibacterium prausnitzii*, whilst propionate is produced by *Bacteroides* species, Negativicutes, and also some *Clostridium* species. Metagenomic screening of more than 3000 sequenced bacterial genomes identified many other species containing butyrate production pathways, with no consistency within families [19]. Since the production of SCFA is not defined by bacterial phylogeny, different methods targeting key genes are required to enumerate bacteria with specific metabolic activities. Louis and co-workers identified two main routes of butyrate production [20], and three pathways for propionate production [18], amongst the colonic microbiota. The primers designed against key metabolic genes in these pathways can help to enumerate functional groups of bacteria in different cohorts. This approach may prove more useful than the current focus on the 16S rRNA gene, which provides information about the bacterial composition but indicates nothing about fluctuations in metabolic activities.

It is important to note that propionate and butyrate are also formed from peptide and amino-acid fermentation by certain Bacteroidetes and Firmicutes species [21]. In vitro studies indicate that aspartate, alanine, threonine, and methionine are the main sources of propionate, whereas butyrate is predominantly derived from fermentation of glutamate, lysine, histidine, cysteine, serine, and methionine.

A targeted gene approach revealed that most bacteria either had the capability to produce propionate or butyrate—very few had genetic capacity to produce both [18]. Some bacteria can, however, alter their fermentation and produce different SCFA under different, substrate-dependent, growth conditions. *Roseburia inulinivorans* is a butyrate producer, but during growth on fucose, it is able to completely change its gene expression pattern, switching on a set of genes capable of utilizing fucose as an energy source, and producing propionate and propanol via a propanediol utilization pathway [22]. *Ruminococcus obeum* produces acetate, formate, and lactate during growth on glucose on pure culture, but additionally produces propionate during growth on fucose, again using the propanediol utilization pathway [18]. Fucose is a particularly important alternative dietary substrate, since many of the epithelial glycoconjugates are fucosylated. The ability of a bacterium to flick a metabolic switch and change its metabolism, and metabolic products, may give the bacterium a competitive advantage during times of low substrate availability. In *Bacteroides thetaiotaomicron*, the presence of fucose as a growth substrate not only stimulates expression of genes involved in fucose metabolism, but intracellular fucose levels are also critical in activating a signalling mechanism to the host, increasing synthesis of fucosylated glycans [23] and thus ensuring a continued supply of substrate to the bacterium. This entire alternative metabolism is upregulated during periods of nutrient depletion, and it may also be important in early colonization events in the infant gut [24].

Altering the carbohydrate content of the diet can also alter the faecal SCFA profile by affecting the bacterial composition. Reducing the carbohydrate content of the diet significantly reduced both faecal butyrate concentrations and numbers of the *Roseburia*/*E. rectale* group in human studies [25], while wheat bran supplementation (consisting of >70% arabinoxylan oligo-saccharides; AXOS) increased the abundance of all three predominant SCFAs and thus also total SCFA concentrations [26]. However, it is probable that the indiscriminate increases in faecal SCFA concentrations observed in studies where the fibre content of the diet is increased are at least partly caused by the increased faecal bulking and reduced transit time resulting in decreased colonic absorption of SCFAs. The FODMAP diet, a diet low in Fermentable Oligo-saccharides, Disaccharides, Monosaccharides And Polyols, and thus designed to reduce large intestinal bacterial fermentation, is increasingly used as an effective therapy to treat IBS. Although the diet is associated with increased faecal pH, presumably due to less bacterial fermentation, the actual faecal concentration of different SCFAs is similar to the control diet [27], illustrating the complex association between SCFA production, absorption, and excretion. Total numbers of bacteria declined on the FODMAP diet compared to the habitual Australian diet, with the proportion of a few specific bacterial groups significantly affected [27].

### Bacterial gas production in the intestinal tract

Gas is an inevitable product of microbial fermentation in anaerobic ecosystems, including the alimentary tract. For example, hydrogen is an important fermentation intermediate and interspecies hydrogen transfer occurs when electron flow shifts from reduced organic products towards proton reduction. This can be achieved through the production of further gases like H_2_S or methane. This disposes of excess reducing power generated in reactions involving the oxidation of organic material.

Gas formation is not a universal trait among bacteria growing anaerobically and the biochemistry of some species involves no gas generation at all [28]. This is the case for common probiotics like lactobacilli and bifidobacteria. It is, therefore, theoretically feasible that probiotic or prebiotic use may reduce gas occurrence in the gut and also help negate odoriferous problems. Gas generated as a consequence of anaerobic bacterial fermentation may be partially excreted via the lungs or as flatus. In the healthy human, investigations have indicated that the volume of flatus excreted can reach up several litres per day [29]. The majority of bacterially generated gas comprises hydrogen, carbon dioxide, and methane, all odourless gases. In general, less than 1% of flatus is oxygen, which together with nitrogen, accounts for only about 26% of flatus [30]. While odoriferous gases constitute less than 1% of total flatus and include NH_3_, hydrogen sulphide, indole, skatole, and volatile amines, their accumulation is certainly noticeable. Key noxious, as well as potentially toxic, constituents are the sulphides, which also act as precursors for other S-based components like mercaptans.

#### Hydrogen

The hydrogen composition of flatus ranges up to 40% and it seems that it is exclusively of microbial origin [31, 32]. Hydrogen is produced by a variety of gut bacteria, the most predominant including *Bacteroides* and *Clostridium*. These genera are common components of the microbiota, implying that they are the principal source of microbial gas [33]. The theoretical rate of hydrogen production far exceeds what is actually excreted, as it can be re-utilized by the gut microbiota. The removal of hydrogen allows a more complete oxidation of organic substrates and, therefore, a higher energy yield from anaerobic fermentation.

There are three main microbial routes by which hydrogen can be removed to enable a depletion of electron sink products such as lactate, succinate, and ethanol, and allow more efficient energy recovery from organic substrates. These are dissimilatory sulphate reduction, methanogenesis, and acetogenesis.

Dissimilatory sulphate reduction is carried out by sulphate reducing bacteria (SRB). These microorganisms utilize sulphate (as opposed to oxygen used in the conventional aerobic respiration) as an electron acceptor for the dissimilation of organic compounds and hydrogen [32]. The main genus of gut SRB is *Desulfovibrio* [31]. Sulphate can be provided in the diet or released following microbial metabolism of sulphated mucins. These are glycoproteins that line the gastrointestinal tract, acting as lubricant as well as a protective barrier between the mucosal surface and the luminal contents.

The utilization of hydrogen to reduce sulphate to sulphide has effects on overall colonic gas production by reducing the amount of free hydrogen in the colon, thereby helping to prevent excessive gas build up:

$${\text{4}}{{\text{H}}_{\text{2}}}+{\text{ S}}{{\text{O}}_{\text{4}}}^{{\text{2}} - }+{\text{ }}{{\text{H}}^+} \to {\text{H}}{{\text{S}}^ - }+{\text{ 4}}{{\text{H}}_{\text{2}}}{\text{O}}.$$

However, the highly toxic nature of the hydrogen sulphide that is generated can have pathological consequences for the host.

Methanogenesis is a further mechanism of hydrogen disposal in the colon, also reducing overall gas accumulation, which is carried out as follows:

$${\text{4}}{{\text{H}}_{\text{2}}}+{\text{ C}}{{\text{O}}_{\text{2}}} \to {\text{C}}{{\text{H}}_{\text{4}}}+{\text{ 2}}{{\text{H}}_{\text{2}}}{\text{O}}.$$

Methanogens and sulphate reducers thus compete for hydrogen in the gut and the process that dominates is dependent on the amount of sulphate available [34, 35]. When sufficient sulphate is available SRB out-compete methanogens for hydrogen due to their greater substrate affinity. While methanogenesis and dissimilatory sulphate reduction are the principle means by which hydrogen is utilized, when either of these mechanisms are in play acetogenesis (the third mechanism of hydrogen utilization) is also feasible. In terms of host health, acetogenesis is likely to be the most favourable mode of hydrogen recycling. The reason for this lies in the fact that, in this process, carbon dioxide and hydrogen are converted into acetate with no evolution of gas [33]:

$${\text{4}}{{\text{H}}_{\text{2}}}+{\text{ C}}{{\text{O}}_{\text{2}}} \to {\text{C}}{{\text{H}}_{\text{3}}}{\text{COOH }}+{\text{ 2}}{{\text{H}}_{\text{2}}}{\text{O}}.$$

However, this reaction is energetically less favourable than dissimilatory sulphate reduction or methanogenesis.

#### Carbon dioxide

Carbon dioxide is another quantitatively significant gas that is expelled in flatus. Carbon dioxide can account for between 5 and 50% of the total flatus volume and as shown above is recycled with hydrogen via methanogenesis and, to a lesser extent, acetogenesis [36].

In contrast to hydrogen and methane, carbon dioxide can be generated by a number of processes, not just bacterial metabolism. Three potential sources of carbon dioxide include its diffusion from the blood into the colonic lumen, the acidification of bicarbonate in the upper gastrointestinal tract, and bacterial metabolism [30]. Some species of clostridia (e.g., *C. sporogenes, C. butyricum*, and *C. perfringens*) produce both carbon dioxide and hydrogen in their metabolic pathways.

#### Clinical aspects

Gas production by the colonic microbiota can exert clinical consequences for the host. For example, a common feature of IBS is excessive gas production and flatus, and is associated with bloating and abdominal distension. An absence of bacterial hydrogen recycling can lead towards pneumatosis cystoides intestinalis which is characterized by excessive gas production and the presence of gas filled cysts on the colonic wall [37]. In this instance, an absence of SRB, methanogenic bacteria, and acetogens causes the individual to produce between 5 and 10 times more gas than is usual.

Recycling of hydrogen via dissimilatory sulphate reduction generates hydrogen sulphide, which is a cell signalling molecule of emerging physiological importance [38], but also is highly toxic to colonic cells and is potentially implicated in inflammatory bowel disease, since sufferers of ulcerative colitis have a universal carriage of SRB [39, 40]. The presence of methane in the colon has been linked with colorectal cancer, although the association may be a consequence of the disease rather than causal, since patients with the condition have slower colonic transit times [41]. This would assist growth of methanogens in the gut due to their slow growing nature. Individuals with lactose intolerance have increased gas production, since the defective absorption of lactose in the upper GIT means that lactose reaches colonic bacteria, and is fermented forming gas.

## Proteins

Early work with human gut contents by Macfarlane and Cummings [42] showed that the colonic microbiota has considerable proteolytic power, converting ingested dietary protein and endogenous protein from host enzymes, mucin, and sloughed off intestinal cells into shorter peptides, amino acids and derivatives, short and branched-chain fatty acids, and gases, including ammonia, H_2_, CO_2_, and H_2_S [42]. This early work was limited at the time to culture-based microbiology techniques, but the authors identified *Bacteroides* and *Propionibacterium* species as the predominant proteolytic species in faecal samples, with proteolysis common also amongst clostridia, streptococci, staphylococci, and *Bacillus* species. Gibson et al. [43] showed that the proteolytic activity of the faecal microbiota differed, both in quantity and quality of protein degradation, from that in the ileum. Faecal proteolyis was more efficacious at degrading the highly globular protein bovine serum albumin, despite having lower overall proteolytic activity compared to ileal effluent. In 1996, the Macfarlane team also provided some of the only information on the metabolic processes governing amino-acid fermentation by gut bacteria using both pure cultures of intestinal bacteria and *in vitro* gut models inoculated with human faeces [44]. They successfully characterized dissimilatory aromatic amino-acid fermentation by these bacteria and measured their production of phenols and indoles upon fermentation of aromatic amino acids using GC-MS. They also measured the impact of pH, carbohydrate availability, and gut model retention time on this activity, and found a preference for amino-acid fermentation at higher ranges of colonic pH and a 60% reduction in this fermentation and end product production (phenols and indoles) when fermentable carbohydrate was available [44]. In seminal work involving intestinal contents from two sudden death victims, Macfarlane et al. [7] reported the metabolic potential of different regions of the colon. The proximal colon was predominantly saccharolytic by nature, whereas protein fermentation increased distally, as did pH, through the transverse colon and into the distal colon. This protein fermentation was associated with increased concentrations of branched-chain fatty acids, phenol, and indole derivatives of amino-acid fermentation and ammonia.

Recently, evidence has emerged that aromatic amino acids (phenylalanine, tyrosine, and tryptophan) can be fermented to phenylpropanoid metabolites, phenylacetic acid, and 4-hydroxyphenyl-acetic acid, which are abundant in faeces [45]. The organisms involved include several species of *Bacteroides, Eubacterium hallii*, and *Clostridium barlettii*. Interestingly, these phenolic compounds are the same as those generated by microbial breakdown of plant polyphenols.

**Fig. 2:**
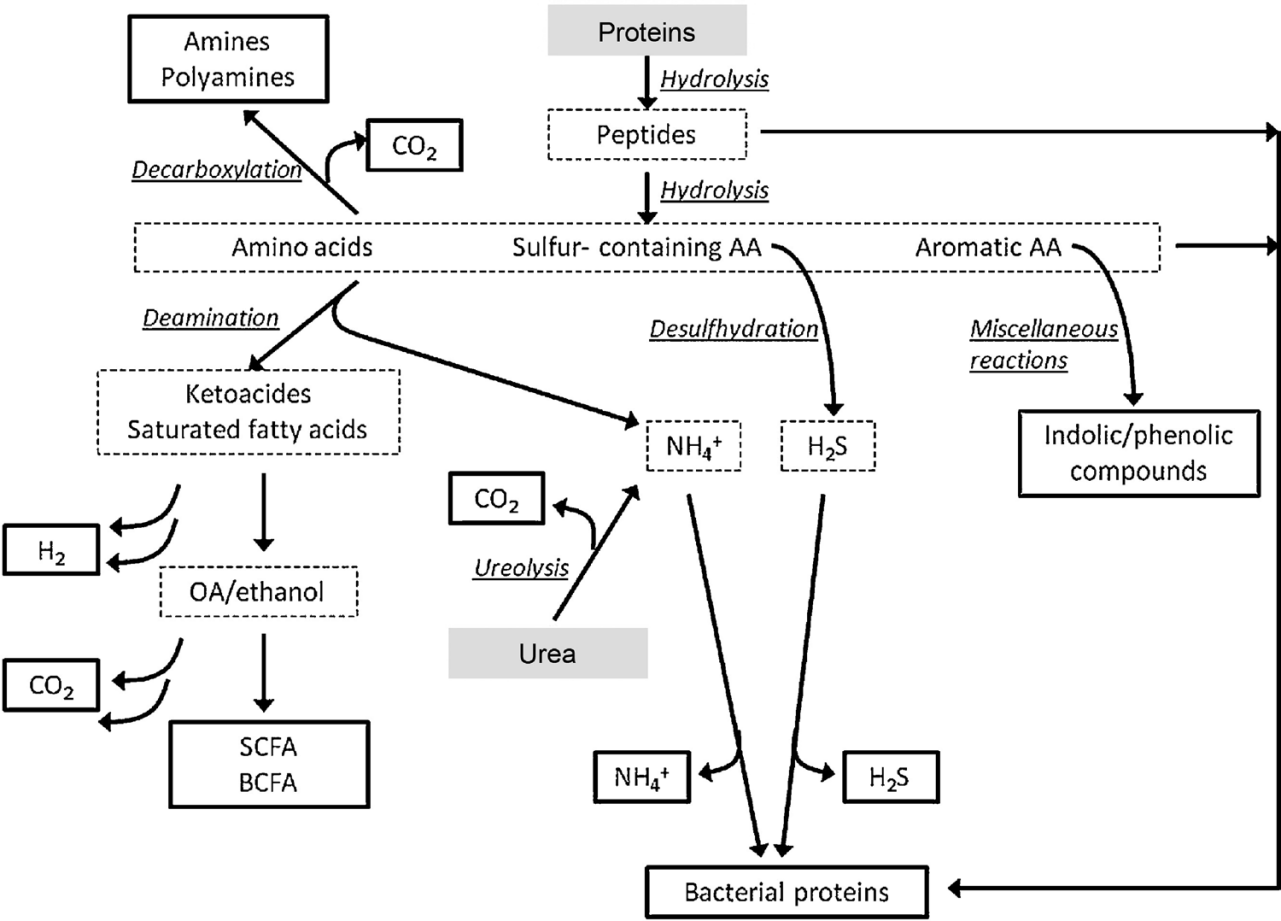
Pathways of gut microbial protein degradation [46]

The complexities of amino-acid utilization and subsequent availability to the host are now becoming apparent and warrant more in-depth scrutiny in specifically designed mechanistic studies (Fig. 2; [46]). Dai et al. [47] showed that bacterial conversion of free amino acids into polypeptides contributes considerably to amino-acid metabolism and bioavailability in the mammalian gut. They also found that the relative concentrations of different amino acids available to intestinal bacteria can impact greatly on overall amino-acid utilization at the community level [48]. For example, they found that L-glutamine regulates small intestinal bacterial metabolism of arginine, serine, and aspartate, and reduced the catabolism of essential and non-essential amino acids. This is especially relevant given the fact that modern food processing has a dramatic effect on the relative concentrations of amino acids present in commonly consumed processed foods and recent evidence for important physiological roles for both essential (e.g., tryptophan) and non-essential amino acids in mammalian nutrition [49–51].

## Vitamin synthesis

It has been known for over 40 years, via studies in germ-free and the conventional rodents and in human volunteers, that the gut microbiota can synthesize certain vitamins, notably vitamin K, and B group vitamins including biotin, cobalamin, folates, nicotinic acid, panthotenic acid, pyridoxine, riboflavin, and thiamine [52]. These vitamins are clearly important for bacterial metabolism, but there is evidence for the metabolic and physiological significance of some of these pathways in mammals. For example, germ-free rats reared without a dietary supplement of vitamin K have low prothrombin levels and develop haemorrhages, while their conventional counterparts have normal prothrombin levels and normal clotting activity [53]. Furthermore, human subjects on low vitamin K diets for 3–4 weeks did not develop vitamin deficiency, but those treated with a broad-spectrum antibiotic to suppress the microbiota showed a significant decrease in plasma prothrombin levels [54]. Metagenomic sequencing has recently been used to provide insight into pathways for vitamin synthesis by the gut microbiota. Le Blanc et al. [55] explored the metabolic potential of gut microbial sequences from two subjects and found that their microbiomes were enriched for a variety of clustered orthologous groups (COGs) involved in the synthesis of deoxyxylulose-5-phosphate, a precursor of thiamine and pyridoxal.

Magnusdottir et al. [56] have systematically explored the genomes of 256 common gut bacteria for the presence of biosynthetic pathways for eight B vitamins, namely biotin, cobalamin, folate, niacin, pantothenate, pyridoxine, riboflavin, and thiamin. This allowed the authors to predict the proportion of each phylum containing potential producers of each vitamin. Some genomes contained all eight pathways, others none. The most commonly synthesised vitamins were riboflavin (166 potential producers) and niacin (162 producers). For riboflavin and biotin, virtually all microbes from the phyla Bacteroidetes, Fusobacteria and Proteobacteria possessed the necessary pathways, with a much smaller proportion of the Firmicutes and Actinobacteria having the potential for vitamin B biosynthesis. In the case of vitamin B12, all the Fusobacteria, compared with 10–50% of the other four phyla were predicted to be producers. Overall, Bacteroidetes appeared to be the phylum with the greatest number of predicted B vitamin producers. Excluding vitamin B12, over 90% of Bacteroidetes were predicted to be producers.

Interestingly, the authors identified several pairs of organisms whose vitamin synthesis pathway patterns complemented each other [56]. This implies cross-feeding between gut microbes, providing essential vitamins for growth. This, in turn, suggests that a major proportion of the microbially produced vitamins are utilized by other non-vitamin producing bacteria. Such utilization limits their availability for the host. The authors estimated the percentage of human daily reference intake of each vitamin obtained from the gut bacteria [56]. Of the eight studied, without considering bacterial utilization, the gut microbiota were estimated to contribute over a quarter of the suggested dietary intake for four vitamins (Table 1, [56]). In addition, there is evidence from studies using a various human and animal colon preparations that the colonic epithelium can absorb a range of B vitamins, including folate, riboflavin, biotin, niacin and thiamine, via specific carrier-mediated mechanisms [57].

**Table 1:** Estimated maximal % of daily reference intake (DRI) of B vitamins that could be provided by the gut microbiota (from Magnusdottir et al. [56])

Vitamin	Intracellular concentration (mmol/gDW)	DRI^a^ (mg/day)	HGM_ratio_	%DRI from HGM
Biotin	9.0 × 10^− 7^	0.03	0.40	4.5
Cobalamin	8.5 × 10^−8^	0.0024	0.42	31
Folate^b^	5.0 × 10^−5^	0.4	0.43	37
Niacin^b^	3.3 × 10^−3^	15	0.63	27
Pantothenate	2.3 × 10^−6^	5	0.51	0.078
Pyridoxine^b^	5.8 × 10^−4^	1.3	0.50	86
Riboflavin	9.0 × 10^−6^	1.2	0.65	2.8
Thiamin^b^	8.7 × 10^−6^	1.15	0.56	2.3

^a^Dietary reference intakes (Standing Committee on the Scientific Evaluation of Dietary Reference Intakes and Its Panel on Folate, Other B Vitamins, and Choline, 1998). Values averaged for male and female references intakes (ages 19–50)

^b^Atomic mass for dihydrofolic acid, nicotinic acid, pyridoxine 5′-phosphate, and thiamine monophosphate

## Bile acids

**Fig. 3:**
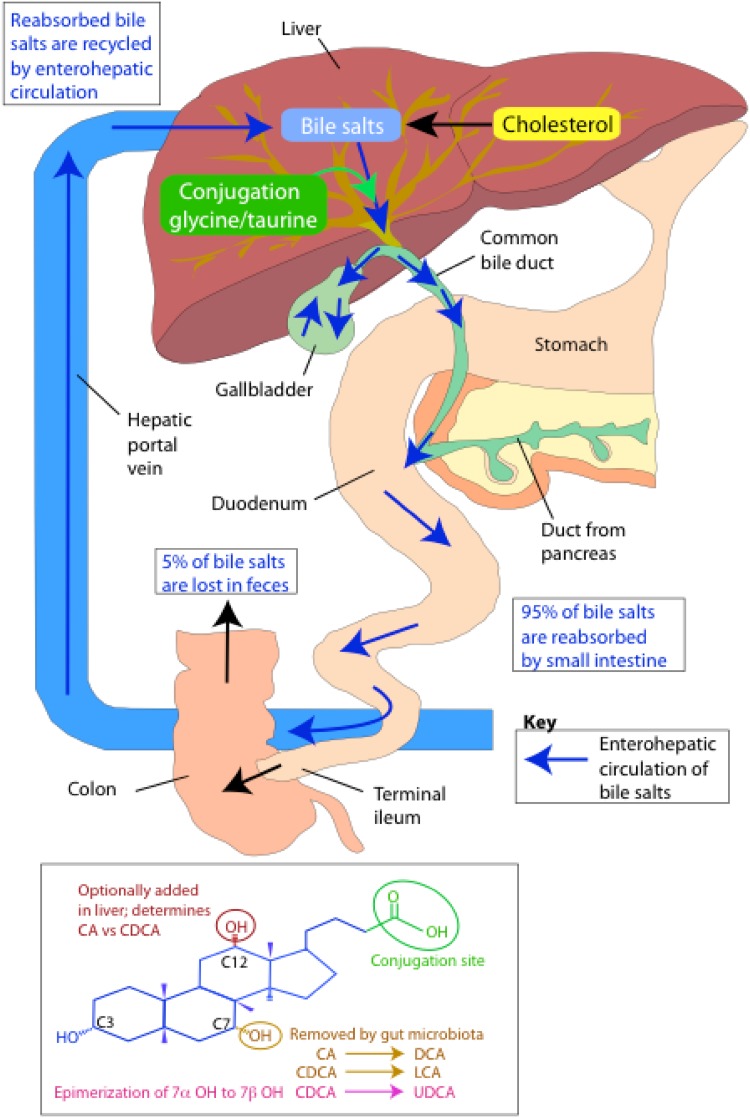
Bile acid metabolism (adapted from [167])

Bile acids are classical examples of trans-genomic metabolites arising from the interactive metabolism between the host genome and the gut microbiome. As outlined in Fig. 3, bile acids are synthesised in the liver from cholesterol to form the two primary bile acids cholic acid (CA) and chenodeoxycholic acid (CDCA). Prior to secretion into bile, *N*-acyl amidation occurs conjugating the carboxyl group of the bile acids to a molecule of either taurine or glycine. This conjugation step produces a molecule that is fully ionised at physiologic pH, enhancing the amphipathic nature and, therefore, detergent properties of the molecule. Upon ingestion of a meal, bile acids stored in the gall bladder are secreted into the small intestine to facilitate lipid digestion and absorption. While the majority of bile acids are actively absorbed in the distal ileum and recycled back to the liver, a small fraction (1–5%; 200–800 mg daily in humans) escapes this enterohepatic circulation and enters the colon. It is here that a bidirectional relationship exists between the gut microbiota and the bile acids. The colonic microbiota are able to modify the structure and properties of the bile acids, while the bile acids possess antimicrobial characteristics and can exert selection pressures on the community structure of the gut microbiota. These characteristics include detergent effects on bacterial cell membranes and the ability to induce DNA damage and disruption to protein structures [58–60]. Interestingly, the potency of deoxycholic acid (DCA), a microbially derived secondary bile acid, is tenfold greater than that of its precursor, CA, due to its greater detergent properties [61]. Hence, greater microbial interaction with the enterohepatic circulation enhances its antimicrobial properties, potentially providing a feedback mechanism to control bacterial populations.

Microbial biotransformation of bile acids includes modification to both the side chain and the steroid nucleus (Fig. 3). At the side chain, a number of gut bacteria possess bile salt hydrolase (BSH) enzymes that are capable of hydrolysing the amide bond between the bile acid and its conjugated amino acid [62]. BSH genes have been identified in the main bacterial genera of the microbiota including *Bacteroides, Bifidobacterium, Clostridium, Lactobacillus*, and *Listeria* [63], and most hydrolyse both glyco and tauro-conjugates. This deconjugation step provides bacteria with a mechanism to reduce the toxicity of the bile acids and is a source of nitrogen, sulphur and carbon atoms [64, 65]. Deconjugated bile acids can be absorbed and returned to the liver for re-conjugation before re-entering the enterohepatic circulation or undergo further bacterial processing.

At the steroid nucleus, a range of microbially mediated modifications can occur resulting in secondary bile acids. Following deconjugation, the C7 hydroxyl group of the bile acid becomes available for microbial dehydroxylation. Genera such as *Clostridium* and *Eubacterium* transform CDCA and CA to the secondary bile acids lithocholic acid (LCA) and DCA, respectively [66]. This 7α dehydroxylation activity is thought to provide these bacteria with an ancillary electron acceptor [65, 67]. These secondary bile acids are potentially cytotoxic for the host and have been associated with colon cancer and cholesterol gallstone formation [68, 69]. To reduce their toxicity, these secondary bile acids undergo further processing in the liver. The inability of the liver to re-hydroxylate secondary bile acids preserves the diversity of the bile acid pool instead, and secondary bile acids are detoxified through conjugation with glycine or taurine, and in some instances sulphate [70]. Another bacterial modification to bile acids is the epimerization of hydroxyl groups from the α to β orientation. Ursodeoxycholic acid (UDCA) is the most common secondary bile acid produced through this action following the epimerization of the 7α hydroxyl group on CDCA [71]. This action decreases the toxicity of the bile acid producing a more favourable microenvironment for the bacteria.

Overall, the circulating and hepatic bile acid pool contains more than 30 known bile acids and the gut microbiome is responsible for driving the majority of this diversity [72]. Variation in bile acid composition has potential to modulate the physico-chemical properties of the overall pool. Bacterial deconjugation reduces the efficiency of bile acids for the emulsification of dietary lipids and micelle formation. This can modify the digestive function of the host as bile acids have key roles facilitating the absorption of dietary lipids, nutrients, and lipid-soluble vitamins. Bile acids are also recognised as important signalling molecules serving as ligands for the nuclear receptor farnesoid X receptor (FXR), and the plasma membrane bound GPR TGR5 [73, 74]. Through binding to these receptors, bile acids can regulate genes critical to their synthesis, conjugation, transport, and detoxification [75–77] as well as lipid [78, 79] and glucose metabolism [80, 81] and energy homeostasis [82]. Variation in the bile acid signature induced by the gut microbiota can, therefore, have downstream effects on a range of host metabolic processes. The global signalling function of bile acids throughout the host metabolic system is suggested by the expression of receptors, transporters, and tissue-specific bile acid signatures outside of the enterohepatic circulation, including in the kidney and heart [83]. These observations demonstrate the systemic regulatory role of bile acids providing a biochemical bridge for the gut microbiome to influence the metabolic status of the host.

## Phytochemicals/polyphenols

Polyphenols from fruits and vegetables are the subject of intensive research due to their putative bioactivities and their relatively high intake levels, about 820 mg/day [84]. Most polyphenols are poorly absorbed in the small intestine and pass into the colon [85]. Studies in germ-free and human microbiota-associated animals and in vitro faecal incubations provide evidence that parent polyphenols are extensively metabolized by the colonic microbiota, which can affect their bioactivity [86, 87].

Polyphenols exhibit structural diversity, which impacts on bioavailability, metabolism, and bioactivity [85]. The main groups comprise phenolic acids, flavonoids (flavonols, flavones, isoflavones, flavanones, anthocyanidins, and flavonols), stilbenes, lignans, and secoiridoids (Table 2, [88]). Most polyphenols are present in food as glycosides (especially flavonoids), i.e., conjugated to various sugars including glucose, galactose, rhamnose, and rutinose. The hydroxycinnamic acids are usually esterified with sugars, organic acids, or lipids. Other polyphenols such as proanthocyandins and ellagitannins are in the form of high molecular weight oligomers and polymers. These conjugated and polymeric forms are generally poorly bioavailable and must be converted to aglycones before absorption. Although for some glucosides, this can be catalysed by intestinal mucosal enzymes, the majority of conjugates, and esters are not absorbed and pass into the colon where they are hydrolyzed by the colonic microbiota [88]. Microbial species involved in hydrolysis include *Bacteroides distasonis, Bacteroides uniformis, Bacteroides ovatus, Enterococcus casseliflavus, Eubacterium cellulosolvens, Lachnospiraceae CG19-1*, and *Eubacterium ramulus* [88, 89].

**Table 2:** Polyphenol metabolism by gut microbiota (after Marin et al. [88])

Polyphenol group	Examples	Principal metabolites	Microbial types	Reference
Phenolic acids				
Benzoic acids	Gallic acid Ellagitannins	Urolithins A & B, isourolithins A & B	*Gordonibacter urolithinfaciens, Gordonibacter pamelaeae*	[157, 158]
Hydroxycinnamic acids	Chlorogenic acid Caffeic acid Ferulic acid	3-(3,4-dihydroxyphenyl)-propionic acid 3-(3-hydroxyphenyl)-propionic acid 3-(4-hydroxyphenyl)-propionic acid Hydroxyphenyl-ethanol Phenylacetic acidBenzoic acid	*Escherichia coli, Bifidobacterium lactis, Lactobacillus gasseri*	[90, 97, 159]
Flavonoids				
Flavonols	Kaempferol Quercetin Myricetin	2-(4-Hydroxyphenyl)propionic acid 3-(3,4-Dihydroxyphenyl)-acetic acid 2-(3-Hydroxyphenyl)acetic acid 3-(3,4-Dihydroxyphenyl)propionic acid 3-(3-Hydroxyphenyl)propionic acid 2-(3,5-Dihydroxyphenyl)acetic acid 2-(3-Hydroxyphenyl)acetic acid	*Clostridium orbiscidens* *Clostridium orbiscidens, Eubacterium.oxidoreducens* *Eubacterium ramulus* *Enterococcus casseliflavus* *Clostridium orbiscidens, E. oxidoreducens*	[90]
Flavanones	Naringenin	3-(4-Hydroxyphenyl)propionic acid 3- phenylpropionic acid	*Clostridium* strains *Eubacterium ramulus*	[85, 90]
	Isoxanthohumol (from hops)	8-Prenyl-naringenin	*Eubacterium limosum*	[160]
Flavan-3-ols	Catechin	3-(3-Hydroxyphenyl)propionic acid 5-(3′,4′-Dihydroxyphenyl)--valerolactone	*Clostridium coccoides, Bifidobacterium infantis*	[88]
	Epicatechin	5-(3,4-Dihydroxyphenyl)valeric acid 3-(3,4-Dihydroxyphenyl)propionic acid		
	Epigallocatechin	5-(3′,4′-Dihydroxyphenyl)--valerolactone 5-(3′,5′-Dihydroxyphenyl)--valerolactone		
Flavones	Luteolin Apigenin	3-(3,4-Dihydroxyphenyl)-propionic acid 3-(4-hydroxyphenyl)-propionic acid 3-(3-hydroxyphenyl)-propionic acid, and 4-hydroxycinnamic acid, phloretin	*Clostridium. orbiscindens, Enterococcus avium* *Eubacterium ramulus Bacteroides distasonis*	[161]
Isoflavones	Daidzein	Equol *O*-desmethylangolensin	Bacteroides ovatus, Streptococcus intermedius, Ruminococcus productus Eggerthella sp.Julong 732, Slakia isoflavoniconvertens, *Slakia equolifaciens, Adlercreutzia equolifaciens* Consortium of *Lactobacillus mucosae Enterococcus faecium Finegoldia magna, Veillonella sp* *Clostridium spp*. HGHA136, *Eubacterium ramulus*	[88, 96, 98, 102, 162, 163]
	Genistein	6′-OH-*O*-desmethylangolensin 2-(4-hydroxyphenyl)propionic acid	Eubacterium ramulus	[163]
Anthocyanidins	Cyanidin Pelargonidin Malvidin	3,4-Dihydroxybenzoic acid 3-hydroxycinnamic acid 4-Hydroxybenzoic acid 3,4-Dimethoxybenzoic acid	*Clostridium saccharogumia Eubacterium ramulus*	[164]
Lignans	Secoisolaricinresinol diglucoside	Enterodiol Enterolactone	*Bact. distasonis, Bact. fragilis, Bact. ovatus, Clostridium cocleatum, Clostridium.sp SDG-MT85-3Db, Butyribacterium methylotrophicum, Eubacterium callanderi, Eubacterium limosum, Peptostreptococcus productus, Clostridium scindens* *Eggerthella lenta, ED-Mt61*/*PY-s6*	[92]
Secoiridoids	Oleuropein Ligstroside	Tyrosol Hydroxytyrosol	Not studied	[165, 166]

**Fig. 4:**
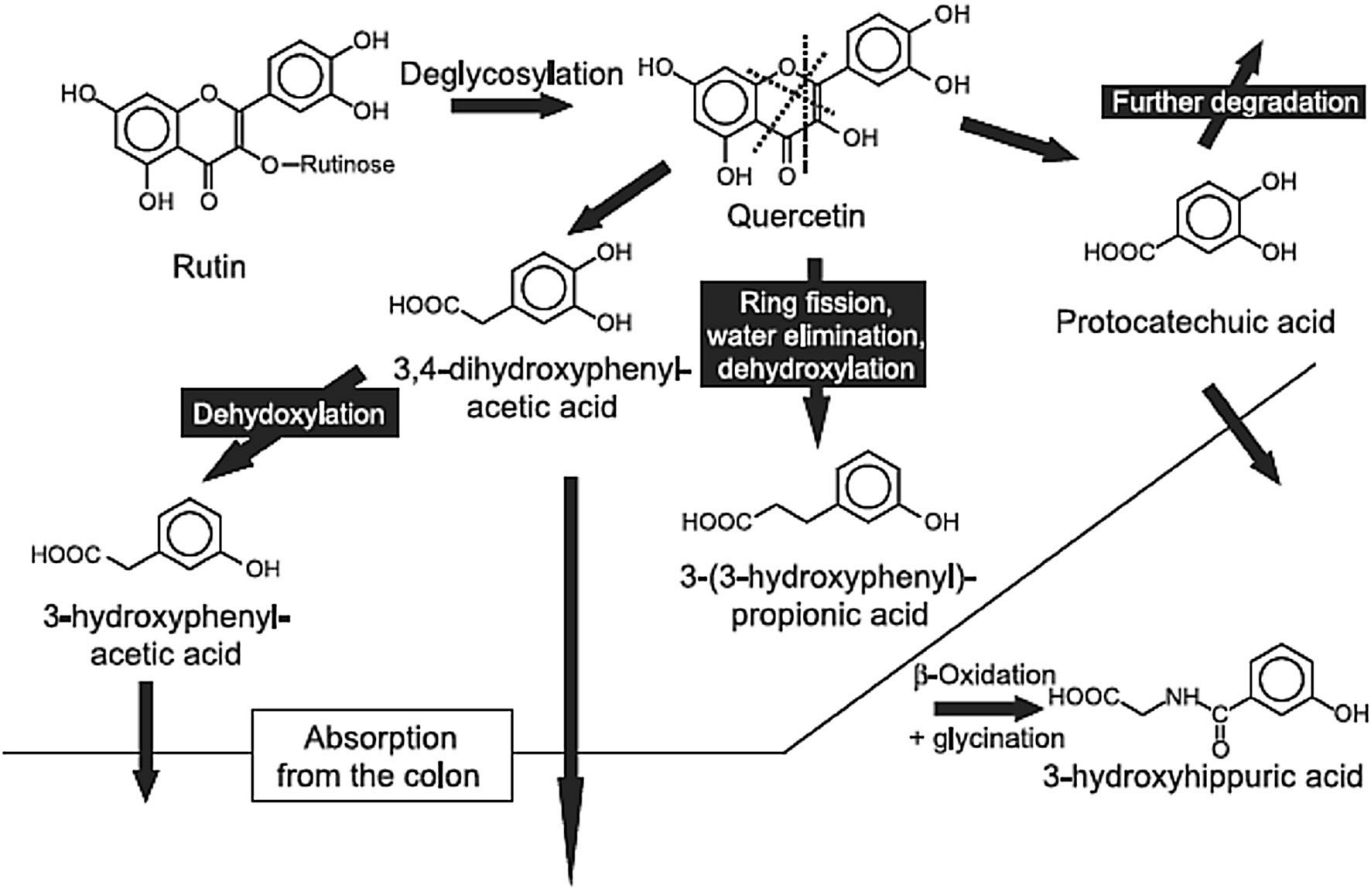
Scheme of gut microbial degradation of rutin [90]

Once the polyphenols have been metabolized to their aglycones or the polymers have been converted to monomers, they are extensively degraded by other components of the colonic microbiota via dehydroxylation, decarboxylation, and ring breakage ultimately generating simpler phenolic compounds, such as hydroxyphenyl-acetic acids and hydroxyphenylpropionic acids. An example of such a pathway is shown for quercetin (Fig. 4; [90]), but equivalent reactions are seen for other flavonoids, phenolic acids and lignans [89–91]. In Table 1, organisms identified as participating in these reactions are shown. It should also be noted that these phenylacetic- and hydroxyphenyl-acetic acids can also be derived from fermentation of aromatic amino acids ([45]; see “Protein” section).

**Fig. 5:**
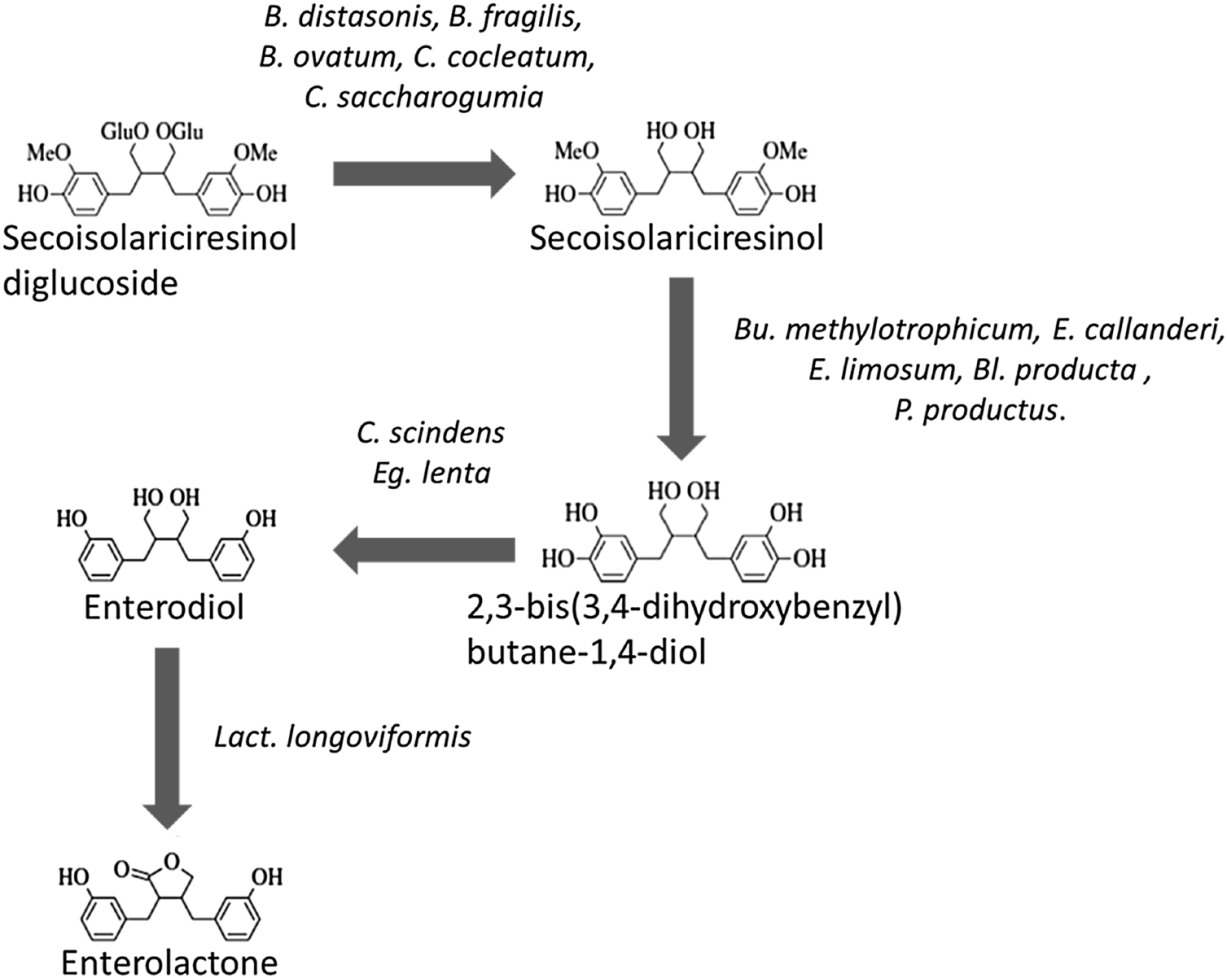
Gut bacterial metabolism of the lignan secoisolariciresinol diglucoside (after Clavel et al. [92]). Abbreviations of bacterial genus names: *B.—Bacteroides; Bu.—Butyribacterium; Bl—Blautia; C.—Clostridium; E.—Eubacterium; Eg.—Eggerthella; Lact.—Lactonifactor; P.—Peptostreptococcus*

It is evident from several studies that the complete metabolism of polyphenol glycosides in the gut requires the involvement of a consortium of microbes. For example, in the case of the lignan, secoisolariciresinol diglucoside, the initial deglycosylation is catalysed by three *Bacteroides* species (*B. distasonis, B. fragilis*, and *B. ovatus*) and two strains of *Clostridium* (*C. cocleatum, C. saccharogumia*). Demethylation of the lignan aglycone involves strains of *Butyribacterium methylotrophicum, Eubacterium callanderi, Eubacterium limosum, Blautia producta, and Peptostreptococcus productus*. Dehydroxylation of secoisolariciresinol is catalysed by *Clostridium scindens* and *Eggerthella lenta* and the final step, dehydrogenation of enterodiol to enterolactone, and closure of the lactone ring is catalysed by subdominant populations of *Clostridiales*, in particular *Lactonifactor longoviformis* (Fig. 5; [92–94]).

Human dietary intervention trials and in vitro faecal metabolism studies with dietary plant polyphenols including flavonoids, isoflavones, lignans, hydroxycinnamic acids, ellagotannins, and anthocyanins have revealed large inter-individual variations in absorption, metabolism, and excretion which have been ascribed to differences in gut microbiota [90, 94–97]. The most well-studied example of such inter-individual variation is the microbial metabolism of the soy isoflavone daidzein which is metabolized by two different pathways depending on the gut microbiota of the subjects [96]. A majority of subjects convert daidzein to *O*-desmethylangolensin with a *Clostridium* species involved. However, about 30% of subjects convert daidzein to (S)-equol via dihydrodaidzein and tetrahydrodaidzein resulting from the activities of a wide range of organisms including *Streptococcus intermedius, B. ovatus, Ruminococcus productus, Eggerthella sp. Julong732, Adlercreutzia equolifaciens, Slakia isoflavoniconvertens*, and *Slakia equolifaciens*. One study [98] found that a consortium of *Lactobacillus mucosae, Enterococcus faecium, and Finegoldia magna EPI3 Veillonella sp*. was sufficient to effect the conversion.

Inter-individual variation is also apparent in the conversion of dietary ellagitannins and ellagic acid to the gut microbial derivatives urolithins. Subjects can be divided into three main phenotypic groups. Group A (25–80% of subjects depending on the trial) produces only urolithin A conjugates, and group B (10–50% of subjects) produces isourolithin A and/or B as well as urolithin A, whereas group 0 (5–25% of subjects) produces no detectable urolithins [97].

There is growing evidence that the metabolism of polyphenols by the microbiota can influence their bioactivity; consequently, inter-individual variation in microbial metabolism could have significance for the health benefits of phytochemicals. Perhaps, the best example is again the soy isoflavone daidzein. There is evidence that equol is more bioactive than its parent isoflavone in a range of areas including oestrogenic and anti-oestrogenic activity, antioxidant capacity and potential anti-cancer effects [99]. Studies of equol producers versus non-producers have suggested that equol production may be important in determining benefits of soy consumption in terms of bone health, menopausal symptoms, and breast cancer, although the data are not consistent [100]. The major metabolites of ellagitannins and ellagic acid, urolithins, are better absorbed than the parent compounds and there is evidence that they are responsible for the health benefits of ellagitannin-containing foods [101]. Consequently, subjects in the phenotypic group 0 (see above) who do not produce urolithins might be expected not to benefit from intake of ellagotannins.

## Methodologies

### Isolation of gut organisms involved in metabolizing dietary components

Studies on the metabolism of the soy isoflavone daidzein to equol provide good examples of methods commonly used to identify specific organisms involved in gut microbiota metabolism of dietary compounds. The identification of equol-producing organisms has been the subject of several studies as a consequence of the potential importance of equol in human health and the intriguing observation that only about 30% of people appear to be capable of its production.

Matthies et al. [102] isolated a novel strain from an equol-producing subject, by serial dilution of a faecal homogenate and incubation in a nutrient broth containing 100 uM daidzein and tetracycline. The latter inhibited the growth of the majority of the faecal microbiota without affecting the metabolism of daidzein. From the highest dilution that contained equol-producing microorganisms, further serial dilutions were prepared and repeated until a pure culture was obtained. On the basis of phenotypic and phylogenetic characterization, the culture was identified as a new species and named *Slackia isoflavoniconvertans*.

In their study to identify equol-producing organisms, Decroos et al. [98] serially diluted a faecal sample from an equol producer and plated on a nutrient agar. Single colonies from the plates were tested for ability to metabolize daidzein. From one such colony a stable, mixed culture capable of converting daidzein to equol was obtained and shown to comprise four bacterial strains identified as *Lactobacillus mucosae, Enterococcus faecium, Finegoldia magna*, and a *Veillonella* sp. The first three were obtained as pure cultures, but interestingly, none was capable of producing equol in pure culture, and the complete consortium was required for the conversion. These isolation attempts illustrate the difficulties that can be experienced in obtaining a pure culture of a bacterium that is intimately dependent on another bacterial species/strain to provide essential growth co-factor(s).

Enrichment techniques have been used extensively in environmental microbiology to isolate organisms capable of degrading contaminants and other xenobiotics in the environment. These techniques usually involve either suspension batch cultures or continuous culture enrichment methods in which the mixed culture is incubated with the xenobiotic as a selection factor, usually as the sole carbon source [103]. These techniques would lend themselves to the isolation of organisms or consortia capable of metabolism dietary compounds, but they have not been widely used in the human gut microbiota area. A recent study by Ziemer [104] illustrates the potential of the technique as applied to the ruminant gut. In this study, continuous culture fermenters containing nutrient medium with cellulose or xylan-pectin as sole carbon sources were inoculated with cattle faeces and run for 8 weeks under operating conditions that modelled the caecum and colon of cattle. Samples were then serially diluted and plated onto carbohydrate-specific agar to isolate colonies that were then identified by 16S rRNA gene sequencing. The communities that arose during the enrichment had a broad microbial diversity representing six phyla (Firmicutes, Bacteroidetes, Proteobacteria, Actinobacteria, Synergistetes, and Fusobacteria). Many of the Firmicutes and Bacteroidetes isolates were related to species demonstrated to possess enzymes involved in fermenting plant cell wall components, but interestingly did not exhibit a high identity to cultured bacteria with sequences in the Ribosomal Database Project and so represented novel genera or species. In fact, over 98% of the isolates were not previously cultured. This methodology, therefore, could provide new opportunities to characterize the metabolic capacities of members of the gut microbiota.

Although the approach of isolating strains capable of metabolizing dietary components provides insight into the potential microorganisms involved in vivo, there are drawbacks, in particular, it clearly focuses only on those gut microorganisms that can be cultured in vitro. Furthermore, the ability of a single strain to metabolize a compound in vitro may not translate into metabolism in the different physico-chemical conditions in the host-gut, and when in the presence of millions of other bacteria, which may be competing for the substrate or acting in partnership to degrade it [105].

### Gut microbial enzyme activity

Much of the focus of recent microbiota research has utilized sequencing methods to describe the composition and relative abundance of the colonic community. Less attention has been paid to the assessment of specific microbial functions, which could be more useful in elucidating the gut metabolism of dietary components and links between the microbiota and health.

Measurement in faecal or colonic samples of the activity of enzymes involved in metabolism of dietary and endogenous compounds has been described for many years. The enzymes β-glycosidase (catalysing the hydrolysis of plant polyphenol glycosides), β-glucuronidase (cleavage of glucuronidated hepatic dietary metabolites), and various polysaccharide-degrading enzymes have been particularly well described [106]. In most cases, this approach of assaying enzyme activities ignores the contribution of individual bacterial types and focuses instead on overall activity in faecal samples. One of the limitations of this approach is that the activities are measured in vitro in faecal suspensions usually using model substrates so may not reflect the activity in vivo where the substrate concentrations and environmental conditions such as pH could be very different.

Studies have also been conducted using a range of gut bacterial isolates to identify the main organisms involved. For example, Dabek et al. [107] screened 40 bacterial strains representative of the main bacterial groups in human faeces for β-glucosidase and β-glucuronidase activity. There was a higher prevalence of β-glucosidase producers (23/40 strains, including most of the *Bifidobacterium* spp. and *Bacteroides thetaiotaomicron* and over half of low G + C% Gram-positive Firmicutes) than β-glucuronidase producers (9/40 strains mainly members of clostridial clusters XIVa and IV). There was also evidence of dramatic strain specificity in β-glucuronidase activity in three *F. prausnitzii* isolates. The study also tested whether exposure to glycoside and glucuronide substrates induced enzyme activity. While there was no effect on most strains, a few exhibited several fold (4–12) increases in activity suggesting that changes in overall faecal enzyme activities in response to dietary exposure may be due to changes in the number of microbes possessing those activities and also enzyme induction in certain strains. McIntosh et al. [108] combined an enzymatic approach and a clone library analysis to study distribution of the β-glucuronidase genes *gus* and BG in the microbiota. Firmicutes accounted for 96% of amplified *gus* sequences, while 59% of BG sequences were attributed to Bacteroidetes.

It should be noted that measurement of enzyme activities of individual strains in vitro does not necessarily reflect activity *in vivo* where the environmental conditions, including pH, and relative abundance of the microbial types may be very different. For example, Cole et al. [105] compared the activity of enzymes measured after in vitro culture and also after the same strains has been introduced into germ-free rats and found significant differences.

More recently, a variety of molecular methods have been exploited to explore enzymatic diversity in the highly complex gut ecosystem. El Kaoutari et al. [109] have designed a custom microarray of non-redundant DNA probes for over 6500 genes coding for enzymes involved in dietary polysaccharide breakdown. It allows the detection of carbohydrate-degrading enzymes present in low abundance bacterial species in the gut. Alternatively, gene-specific primers can be used to enumerate all bacteria capable of performing a specific role in the gut, using qPCR, as has been done for butyrate producing bacteria [17]. However, both these techniques only identify the presence of genes, and not whether they are actively expressed at a specific time.

### Omics approaches

There is a growing awareness of the importance of the gut microbiome in the overall system of the host. This has led to the inclusion of top-down approaches studying the composition and functionality of the microbiota, so-called ‘-omics’ approaches. Metagenomics provides insight into the genes that could be expressed, while metatranscriptomics reveals information about regulatory networks and gene expression and combined with metaproteomics, and metabolomics informs about the functionality of the microbiota and, therefore, provides some strong insights into microbial activities in the gut.

Studies are performed in an unbiased fashion with the focus on hypothesis generation rather than hypothesis testing. This has proven particularly effective for studying the gut microbiota due to the relatively limited understanding of this multi-dimensional dynamic variable. Each ‘-omic’ technology provides its own unique perspective of the microbiota and its impact on the host, so to fully exploit their potential multiple ‘-omic’ approaches can be applied simultaneously and results integrated, preferably from the same sample. With the help of mathematical modelling, this enables a comprehensive understanding of the microbial ecosystem to be gleaned and its contribution to the overall biological system to be studied at the molecular level. This represents a significant technical and bioinformatic challenge, although a new methodological framework developed by Roume et al. [110] for the co-extraction of DNA, large and small RNA, proteins, and polar and non-polar metabolites from single samples of microbial communities represents a significant step in this process.

#### Metagenomics

Metagenomics has extensively been used to investigate differences in microbiota composition in disease states such as inflammatory bowel disease, obesity, and diabetes compared to healthy individuals, but it has also revealed novel changes in microbiota function in some diseases [111]. For example, Wei et al. [112] reported that the faecal microbiota of 20 patients with hepatitis B cirrhosis of the liver showed enrichment of metabolism of glutathione, branched-chain amino acids, nitrogen, lipids, and gluconeogenesis, and a decrease in aromatic amino acids and bile acid-related metabolism in comparison to control subjects.

Metagenomic analysis is being increasingly used to study functional genes of the gut microbiota. Jones et al. [63] used this methodology to study the distribution of BSH genes. Via metagenomic analyses, they identified functional BSH in all the main bacterial divisions and Archaea in the gut and demonstrated that BSH is a conserved adaptation to the amount of conjugated bile acids in the gut and exhibits a high level of redundancy. Of particular relevance to the present review is the approach taken in a recent paper by Mohammed and Guda [113]. The authors developed an ensemble of machine learning methods termed ECemble (Enzyme Classification using ensemble approach) to model and predict enzymes from protein sequences and identify enzyme classes and subclasses at high resolution. The method was then applied to predict enzymes encoded by the human gut microbiome from gut metagenomic samples, and to study the role of microbe-derived enzymes in the human metabolism. They identified 48 pathways that have at least one bacteria-encoded enzyme. The pathways were primarily involved in the metabolism of amino acids, lipids, co-factors, and vitamins. Subsequently, the methods were used to demonstrate differences in the profiles of gut microbiota-derived enzymes in lean and obese subjects and in patients with IBD. For example, the microbiota of obese subjects was enriched in polygalacturonase, which is encoded by *Bacteroide*s and *Prevotella* species. In contrast, urease-encoding bacteria were found in fewer numbers in obese versus lean subjects.

A number of metagenomic studies have focused on so-called carbohydrate active enzymes (CAZymes) due to the critical role that the gut microorganisms play in the breakdown of dietary fibre and other non-absorbed carbohydrates in the gut. Such an approach is not restricted to the study of the enzymatic activity of cultivable microbes and has revealed a wide diversity of CAZymes of at least 81 families of glycoside-hydrolases. For example, Tasse et al. [114] using in-depth pyrosequencing, discovered 73 CAZymes from 35 different families and also identified 18 multigenic clusters encoding complementary enzyme activities for fibre degradation.

Single cell genomics is an emerging technology in which single microbial cells are isolated from a sample, their DNA extracted and amplified and then shotgun sequenced [115]. The advantage of this approach is that genomic data can be placed in a phylogenetic context even where the function of a putative gene is unknown and information from rare or uncharacterized species can be obtained. This has the potential to complement metagenomics by aiding the functional assignment of metagenomic data.

Although metagenomics is a powerful tool for investigating the gut microbiota, it does have limitations. These have been discussed in detail by Wang et al. [111] but include the requirement for sufficient high-quality DNA, the impact of different DNA extraction methods and kits on results, and of particular relevance for functional metagenomics, the limitations in the size and quality of reference databases, which impedes the assignment of functions to the data obtained. Finally, the presence of a gene does not inform us about gene expression patterns. Metatranscriptomics, metaproteomics, and metabonomics enable the latter to be more effectively addressed.

#### Metatranscriptomics

Metatranscriptomics extracts and sequences mRNAs from a microbial ecosystem to determine the genes that may be expressed in that community. It usually involves reverse transcription to generate cDNA, which is then sequenced using similar methodologies as for metagenomics. Metatranscriptomics allows the identification of novel non-coding RNAs, including small RNAs thought to play important roles in biological processes such as quorum sensing and stress response [116]. The approach has mostly been applied to samples from water and soil environments and less frequently to the gut microbiota [117, 118] and the microbiota studies need to be interpreted with considerable caution, given the major limitation of the short half-life of bacterial mRNAs, although this is less of an issue for studies using ribosomal RNAs, which are more stable.

Gosalbes et al. [118] performed a metatranscriptomic analysis of faecal microbiota from 10 healthy subjects. Microbial cDNAs from each sample were sequenced by 454 methodology and analysis of the 16 S rRNA transcripts revealed that Firmicutes and Bacteroidetes were the sources of the greatest number of transcripts (49 and 31%, respectively) with smaller numbers from Proteobacteria (3.7%), Actinobacteria (0.4%), and Lentisphaerae (0.2%). The majority of the Firmicutes sequences fell into the Lachnospiraceae and Ruminococcaceae families, which contain pectin and cellulose degraders. In the Bacteroidetes phylum Bacteroidaceae, Prevotellaceae, and Rickenellaceae families were functionally the most important. Interestingly, the most active families were the same in all the volunteers.

The non-ribosomal transcripts from the faecal samples were searched by BLASTX against an established NCBI COG database to obtain a functional distribution for each sample. The pattern was very similar for all the samples with carbohydrate transport and metabolism, energy production and conversion, and synthesis of cellular components being the main activities. Other areas such as amino acid and lipid metabolism, cell motility, and secondary metabolite biosynthesis were underrepresented in the metatranscriptome. These results are consistent with an earlier, smaller study by Turnbaugh et al. [117] in monozygotic twins in which the genes with higher relative expression included those for carbohydrate metabolism, energy metabolism, nucleotide metabolism, and those associated with essential cell processes, e.g., RNA polymerase and glycolysis.

As with all the ‘-omics’ approaches, metatranscriptomics has its limitations and studies are challenging both technically and in terms of bioinformatics. The short half-life of mRNA leads to difficulty in the detection of short-term responses to environmental changes, consequentially extrapolating results obtained from transcriptional analysis of faecal samples to functions within the large intestine itself can present problems [115, 117].

#### Metaproteomics

Metaproteomics aims to characterize the complete profile of gene translation products and can yield additional information about post-translational modifications and localization over that provided by metatranscriptomics measurements [119]. One of the advantages of metaproteomics is that it is possible to link proteins to specific taxonomic groups, thus providing insight into the microbes at species and strain level involved in specific catalytic functions and pathways, i.e., genotype–phenotype linkages [120].

Methodologies for metaproteomics are in a state of development, but typically they involve heat treatment of the faecal sample and extensive bead beating to extract and denature the proteins, which are subsequently enzymatically digested to peptides. Peptide analysis is usually by nano-2D-LC-MS-MS and COG assignments are determined for each peptide sequence by BLAST against the NCBI COG database. Microbial community functions are analyzed by grouping proteins into COG categories.

Metaproteomic studies on the gut microbiota to date have been performed in small numbers of subjects (usually *n* = 1–3), which limits the conclusions that can be drawn, but the results have shown some consistencies. Verberkmoes et al. [121] conducted a faecal metaproteomic analysis of a pair of adult female monozygotic twins. Analysis was by nano-2D-LC-MS-MS and the proteins identified by database searches were classified into COG categories. In both subjects, the most abundant COG functions were energy production, amino-acid metabolism, nucleotide metabolism, carbohydrate metabolism, translation, and protein folding. The authors compared the metaproteomic profile with a previously published metagenomic profile of two individuals which revealed that in contrast to the most abundant functions identified in the metaproteome above, the metagenome was dominated by proteins involved in inorganic ion metabolism, cell wall and membrane biogenesis, cell division, and secondary metabolite biosynthesis.

Kolmeder et al. [122] investigated composition and temporal stability of the faecal metaproteome in samples collected at 2 time points from 3 healthy subjects over a period of 6–12 months. The results indicated that the faecal metaproteome is subject-specific and is stable over a 1-year period. A stable common core of about 1000 proteins was recognised in each of the subjects. The most abundant core protein was found to be glutamate dehydrogenase, and this enzyme showed high level of redundancy in the intestinal tract, since it was associated with a number of microbial families, *Lachnospiraceae, Bacteroidaceae, Ruminococcaceae*, and *Bifidobacteriaceae*. Other high abundance proteins included pyruvate-formate lyase, which converts pyruvate to acetyl-CoA and formate, and chaperone proteins involved in protein folding and Fe-S cluster formation. About 10% of the total proteome comprised proteins involved in carbohydrate transport and metabolism including ABC sugar transporters and glycolytic enzymes. When the COGs were mapped onto pathways, the main functional categories were metabolism of carbohydrates, nucleotides, energy, amino acids, and co-factors and vitamins (especially B12 and folic acid).

Metaproteomics has also been applied to faecal samples from a lean and an obese subject and to comparisons of Crohn’s Disease patients and healthy subjects (reviewed by Xiong et al. [119]). Young et al. [120] used shotgun proteomics to characterize the functional changes in the faecal microbiota 7–21 days after birth of a preterm infant. The results suggested that the developing microbial community initially focuses its resources on cell division, protein production, and lipid metabolism later switching to more complex metabolic functions, such as carbohydrate metabolism, and secreting and trafficking proteins. It is noteworthy that this functional distribution seen after 3 weeks was similar to that observed in the adult human gut [121].

Metaproteomics is a developing technology and has its limitations, in particular there is no reference protocol, so it can be difficult to compare studies and the bioinformatic systems for metaproteomics are less well developed than those for metagenomics. Kolmeder and de Vos [123] have discussed in detail published methodologies, highlighting the importance of sampling techniques and sample preparation and processing.

#### Metabolic profiling (metabonomics/metabolomics)

Metabolic profiling has emerged as a powerful systems biology approach simultaneously measuring the low-molecular weight compounds in a biological sample, capturing the metabolic profile or phenotype. In the host, these metabolic signatures contain thousands of molecular components that arise from endogenous and exogenous metabolic processes, environmental inputs, and metabolic interactions between the host and environment. The environmental inputs can include dietary components and products of gut microbial activity. A major strength of using metabonomics to study the gut microbiota is the ability to measure metabolites in host samples that derive directly from the microbiome, for example the SCFAs. This provides a direct read-out of gut microbial activity and variations due to diet. Furthermore, upon absorption from the gut, microbial products can enter host metabolic processes resulting in downstream metabolic perturbations and the generation of microbial-host co-metabolites, all of which can be captured by metabolic profiling.

Practically, metabolic profiling can be applied to a range of different sample types and experimental models. It can be used to characterize the metabolites in samples from in vitro experiments, including pure cultures and complex gut models, and various sample types collected from in vivo studies [13]. In vivo samples can include biofluids, such as urine, blood, faecal water, saliva, and cerebrospinal fluid, and various tissue samples such as those collected from the gut, liver and brain. To measure the metabolic profile of a sample, two analytical platforms are typically used, ^1^HNMR spectroscopy and mass spectroscopy (MS). Both techniques are capable of simultaneously capturing quantitative and structural information on a broad range of metabolites in an unbiased manner in a single measurement. Comprehensive reviews on these analytical techniques have been published [124–126]. ^1^H NMR spectroscopy measures protons (^1^H) on metabolites in a sample and MS measures the exact mass of molecular ions in a sample and how they fragment. This information can then be used to identify the metabolites present and their abundance. MS is usually preceded by a separation step to allow the analysis of complex mixtures, which includes liquid or gas chromatography, or in some cases capillary electrophoresis. Although a single analytical technique is routinely applied in metabonomic studies, these two techniques are complementary and their parallel application can provide wide metabolome coverage.

The metabolic phenotype acquired from these techniques is multivariate in nature containing hundreds to thousands of metabolites. To extract latent information associated with gut microbial function or their influence on host metabolism from this multi-dimensional data, a range of pattern recognition techniques are applied [127–129]. Standard multivariate statistical techniques used for metabolic profiling studies include the unsupervised approaches, principal components analysis (PCA), and hierarchical clustering analysis (HCA) and the supervised approach, projection to latent structures (PLS) analysis. Unsupervised methods are concerned with modelling variation within the data and have no a priori knowledge of sample classification. In contrast, supervised methods use known information of the samples (e.g., germ-free versus conventional status; placebo versus prebiotic intervention) to extract information in the metabolic data that are related to this information.

The utility of this approach for studying the microbiome has been demonstrated in animal models of altered microbial status, such as germ-free, gnotobiotic, antibiotic-treated animals, and also in human studies [82, 130–132]. These studies have shown the extensive reach of the gut microbiota throughout the metabolic system of the host and the diverse pathways modulated. This is not just restricted locally to the immediate environment of the gut but systemically to peripheral tissues such as the heart and brain. For example, dietary choline from sources such as red meat and eggs can be metabolized by the microbes in the gut to trimethylamine and dimethylamine. Trimethylamine is toxic and requires oxidation in the liver by the flavin-containing monooxygenase 3 (FMO 3) enzyme before being excreted as trimethylamine-*N*-oxide (TMAO). TMAO can be used as an electron acceptor by *Escherichia coli*, and, interestingly, has been implicated as a risk factor for CVD [133].

A vast amount of information is captured with metabolic profiling and various inherent (e.g., genetic constitution, age, and gender) and environmental (e.g., diet, alcohol intake, and drug therapy) factors can influence the metabolic phenotype. Unrelated variation in these metabolic signatures can often mask or obscure the variation resulting from gut microbial activity. As such, careful study design is essential when investigating the role of the gut microbiota on host metabolism to minimise this unrelated noise. Although this can be tightly controlled in animal studies, it represents a major challenge for human studies. One way to overcome this issue is through the use of statistical approaches. Orthogonal projections to latent structures (OPLS) is one such method applying an orthogonal signal correction (OSC) to remove metabolic variation unrelated to the variable being studied. This improves the interpretation of the data enabling the influence of the gut microbiota to be illuminated. Another limitation in metabolic profiling studies is the metabolite identification stage. Once significant metabolic associations are discovered assigning an identity, pathway and/or function to these features can represent a bottleneck in the metabonomic workflow. Advancements in databases, software platforms, and analytical approaches are helping to overcome these limitations and expedite this process.

#### Stable isotope probing (SIP)

The use of stable isotopes (e.g., ^13^C, ^15^N, and ^18^O) can help elucidate the fate of specific compounds within complex microbial systems such as the gut and can be particularly useful if combined with ‘-omics’ techniques [115]. Tannock et al. [134] used the technique to identify the main microbial users of inulin in the rat gut. Inulin labelled with ^13^C was fed to rats and RNA extracted from caecal contents by isopycnic buoyant density gradients was used to detect labelled RNA from cells that had metabolized the inulin. 16 S rRNA genes amplified from cDNA from the labelled fractions were sequenced and showed that *Bacteroides uniformis, Blautia glucerasea, Clostridium indolis*, and *Bifidobacterium animalis* were the main species utilizing inulin in these rats.

### Use of mathematical modelling to mimic the gut ecosystem

Meta-omic analyses have resulted in a tremendous amount of data on the composition, encoded functionalities, and metabolic output of the human gut microbiota [135]. However, the inherent complexity of the gut ecosystem hinders the interpretation of this wealth of data [136]. A systems-level understanding of the microbiota has to include the underlying complex interactions, since the gut ecosystem as a whole is more than the sum of its parts [136]. A complete, integrated view of the human gut microbiota requires the use of mathematical models.

Identifying novel food ingredients which may have beneficial effects on the gut microbiota when provided as dietary supplements is often difficult due to the large number of variables that need to be compared in well-designed controlled human intervention studies. Experimental models (small animals and fermenter systems) have proved extremely useful, but even they prove time consuming and not all variables can be compared. Mathematical models offer an alternative to try and evaluate bacterial interactions and the impact of different dietary components on microbial composition and activity, at least with the aim of refining the choices to be used in the experimental situation.

One approach applied to simulate the behaviour of gut microbial communities is kinetic modelling. A kinetic model showed the role of bacterial cross-feeding in the conversion of lactate to butyrate by two distinct but abundant human gut bacteria, *Eubacterium hallii* and *Anaerostipes coli* [137]. Kettle et al. [138, 139] created a minimal model of the intestinal ecosystem, distilling the microbiota down into 10 bacterial functional groups, each comprising of a mixture of at least 10 bacteria. Pure and mixed culture data was used to provide assumptions of growth rates, substrate specificity and metabolic activities for each of the functional groups. The model successfully predicted the switch between high butyrate production at pH 5.5 and high propionate production at pH 6.5 that had occurred in a previous continuous flow fermenter experiment [139, 140]. The model was also used to estimate the effect of removing entire functional groups (or single strains within a functional group) on the community profile and activity, revealing changes in both SCFA concentrations and abundances of other groups [139]. Such models could be of huge benefit in assessing the consequence of bacterial species shown to be missing, or extra, in disease states on the development of the disease. They would also show whether simply adding back a ‘missing’ bacterium would be sufficient to potentially change the course of the development of the disease. Such models could also be used to illustrate the potential consequences to microbial composition and metabolite production during periods of starvation or substrate depletion or replacement.

#### Agent-based modelling

Agent-based modelling (ABM) is another approach frequently employed to study the dynamic behaviour of ecological systems in silico. In ABM, objects with well-defined properties (representing, e.g., bacterial cells), are allowed to interact with each other, resulting in a dynamic model that can depict real-time behaviour of a biological system [141]. A recent study used ABM to simulate the positive (e.g., mutualistic) and negative (e.g., competitive) interactions between two bacteria representing a typical Bacteroidetes and Firmicutes, respectively [141]. A simulation of exposure to antibiotics and subsequent recovery confirmed that feedback mechanisms between bacterial species enabled the restoration of system stability after antibiotic perturbation [141].

#### Topological analysis of metabolic networks

A genome-scale metabolic reconstruction summarizes known biochemical reactions of a target organism in a well-structured manner. Several studies have applied metabolic networks of the human gut microbiome, revealing topological properties of its global metabolic network and characterizing the microbiome’s metabolic potential (reviewed in Manor et al. and Heinken and Thiele [136, 142]). Recently, metabolic networks of gut microbes have been integrated with a Boolean dynamic model constructed from time series metagenomic data [143]. The model predicted that the commensal *Barnesiella intestinihominis* can inhibit the growth of *Clostridium difficile*, which was validated experimentally [143]. Another study integrated community-wide metabolic networks with metagenomic and metabolomic data and the community-wide metabolic turnover was subsequently predicted for the vaginal and the gut microbiome [144]. While correlation-based statistical analyses of metabolomic measurements are not mechanistic, this framework has the advantage of proposing mechanisms for the contributions of species to the turnover of particular metabolites [144].

#### Modelling emerging phenotypic properties

The constraint-based reconstruction and analysis (COBRA) approach uses genome-scale reconstructions (GENREs) that are constructed from the genome of a target organism and curated and validated against the available literature [145]. More than 20 GENREs have been built for microorganisms inhabiting the human body [142]. GENREs are converted into mathematical models that are tailored to specific conditions by enforcing constraints: physico-chemical (e.g., mass–charge balance), environmental (e.g., nutrient availability), and regulatory (e.g., gene expression) [145]. By defining an objective, e.g., biomass production, metabolic fluxes through the network that satisfy this objective are predicted [146]. For instance, by imposing constraints on nutrient availability, the growth requirements of a reconstructed organism can be predicted. This approach resulted in the prediction and subsequent experimental validation of a defined medium for *Faecalibacterium prausnitzii* [147]. Moreover, GENREs enable the evaluation of a species’ functional repertoire. In a large-scale study, metabolic networks were retrieved from the genomes of 301 representative human gut microbes and their metabolic capabilities were systematically compared in the context of phylogenetic distance between species [148]. The analysis revealed an exponential relationship between metabolic and phylogenetic distance, with closely related species being more metabolically diverse than could be expected in a linear relationship [148].

Of particular interest is the prediction of *in silico* interactions in microbial communities, and between the gut microbiota and the human host. Similar to the kinetic model described above, such a multi-species model can predict the effect of perturbing community composition (e.g., the removal of key species). Moreover, the effects of varying nutrient environments (e.g., different diets) on the community can be explored. In a first effort to model a host-gut microbe symbiosis, a metabolic model of the mouse was joined with *Bacteroides thetaiotaomicron* and their mutually beneficial cross-feeding was simulated on five dietary regimes [149]. Moreover, the rescue of lethal host gene defects by *Bacteroides thetaiotaomicron* was predicted [149]. The effect of a simplified model gut microbiota on human metabolites was modelled by joining the human reconstruction Recon2 with 11 published, manually curated GENREs of gut microbes from three phyla [150]. The combined contribution of secretion products by microbes and dietary input on the host’s metabolome was quantified on four simulated diets [150]. The underlying mechanisms were predicted for the microbial contribution to host secretion for the examples of glutathione, taurine, and leukotrienes [150].

Constraint-based modelling is commonly applied to the *in silico* prediction of microbe–microbe interactions (reviewed in Heinken and Thiele, Biggs et al., and Zomorrodi et al. [142, 151, 152]). Typically, two or more GENREs are joined to construct community models with well-defined species–species boundaries, thus allowing the prediction of metabolic cross-feeding between species [142]. To investigate the effect of varying metabolic environments on microbe–microbe interactions, 11 published gut microbe GENREs were joined in all pairwise combinations and the outcome (positive, neutral, negative interaction) was predicted [153]. The metabolic exchange and the distribution of resources between the pairs were simulated on four simulated gut microenvironments and three diets [153]. The model predicted that the need to regenerate reducing equivalents enforced mutualism in certain pairs under anoxic conditions [153]. In vitro screens of pairwise interactions between microbes are laborious and the described in silico framework constitutes an important first step for the prediction of candidate pairs of interest that would be subsequently validated experimentally.

GENREs also provide a useful framework for the contextualization of meta-omic data, with a variety of constraint-based methods for the integration of such measurements already available [154].

In summary, important advances have been made in the construction of mathematical models that capture key aspects of the gut microbiota and its interactions with its host, summarize the current knowledge on its metabolism, and propose hypotheses that can be experimentally validated. In future efforts, such models will result in the elucidation of previously unknown, non-intuitive relationships between the gut microbiota and host physiological states. Moreover, dietary and drug interventions to favourably manipulate the human gut microbiota may be predicted in silico prior to experimental validation. Knowledge of an individual’s microbial composition will ultimately enable models to be tailored to predict effects of specific diets or supplements at an individual level.

## Conclusions

The wealth of metabolic functionality encoded within the gut microbiome extends the biochemical flexibility of the host to process a wide range of dietary substrates. Carbohydrate metabolism and transport is clearly a major catalytic function of the microbiota with important consequences for the host, and the metabolic pathways and end products have been well studied and are characterized by great flexibility in response to substrate availability. Similarly, pathways for metabolism of other dietary macromolecules, namely protein, have been elucidated as have those for vitamin synthesis. The microbiota also has extensive capacity to metabolize phytochemicals, particularly polyphenols, by diverse, well-characterized pathways. There is extensive evidence that inter-individual differences in metabolism of dietary polyphenols are largely a consequence of differences in gut microbiota composition and that these can have implications for the effects of some polyphenols on health. It should be noted that the uptake and utilization of microbial metabolites by other members of the microbiota and absorption by the host result in a highly dynamic system of metabolic fluxes and makes determination of changes in concentrations of metabolites with time very difficult using current single snapshot analyses in faecal samples. Application of stable isotope probes in combination with mathematical modelling may prove valuable in this area.

Importantly, there exists a large amount of functional redundancy within the microbiota with different bacteria performing the same or similar functions. BSHs and CAZymes are a good example of this redundancy, being encoded in the genomes of bacteria from several different phyla [62]. As such, compositional variation may not necessarily translate into a functional variation relevant to the host. Furthermore, extensive cross-feeding networks exist within this dynamic ecosystem resulting in a range of possible outcomes for the same substrate depending upon the species present and their proximity. Accordingly, there is a growing appreciation that measuring composition alone is no longer sufficient to gain meaningful insights into the functional status of the gut microbiota, its metabolic interaction with the host, and its potential to modulate host health and disease.

Gut microbiota-derived products can be absorbed from the gut and enter host endogenous and exogenous pathways to influence the overall metabolic phenotype of the host. In addition, metabolites generated by the host can be secreted into the gut via the enterohepatic circulation and serve as substrates for the resident microbes. Collectively, these processes result in a biochemical cross-talk between the host genome and microbiome with the microbiota able to exert a strong influence on the metabolic phenotype of the host. For example, gut microbial BSH enzyme expression in mice altered plasma bile acid signatures and concomitantly the transcription of genes involved in host lipid metabolism and metabolic signalling pathways and also influenced cholesterol metabolism and weight gain [155]. Recent evidence indicates that the SCFAs acetate, propionate and butyrate can play a significant role in central appetite regulation (via hypothalamic neuronal activation patterning and changes in the expression profiles of regulatory neuropeptides) and energy homeostasis via influencing intestinal gluconeogenesis. Further study of these types of interactions is essential to understand how the gut microbiota influence host health and disease.

## Abbreviations

AXOS: Arabinoxylan oligosaccharide
BSH: Bile salt hydrolase
CA: Cholic acid
cAMP: Cyclic adenosine monophosphate
CAzyme: Carbohydrate active enzyme
CDCA: Chenodeoxycholic acid
CE: Capillary electrophoresis
COG: Clustered orthologous group
CVD: Cardiovascular disease
DCA: Deoxycholic acid
FMO: Flavin-containing monooxygenase
FOS: Fructo-oligo-saccharide
FXR: Farnesoid X receptor
GC: Gas chromatography
GIT: Gastrointestinal tract
GPR: G protein-coupled receptor
HCA: Hierarchical clustering analysis
HGM: Human gut microbiota
IBD: Inflammatory bowel disease
IBS: Irritable bowel syndrome
LC: Liquid chromatography
LCA: Lithocholic acid
MS: Mass spectrometry
NMR: Nuclear magnetic resonance
PCA: Principal components analysis
qPCR: Quantitative polymerase chain reaction
PLS: Projection to latent structures
SCFA: Short chain fatty acid
SIP: Stable isotope probing
SRB: Sulphate reducing bacteria
TMAO: Trimethylamine-*N*-oxide
UDCA: Ursodeoxycholic acid
